# Conformational Properties of Active Semiflexible Polymers

**DOI:** 10.3390/polym8080304

**Published:** 2016-08-12

**Authors:** Thomas Eisenstecken, Gerhard Gompper, Roland G. Winkler

**Affiliations:** Theoretical Soft Matter and Biophysics, Institute of Complex Systems and Institute for Advanced Simulation, Forschungszentrum Jülich, D-52425 Jülich, Germany; t.eisenstecken@fz-juelich.de (T.E.); g.gompper@fz-juelich.de (G.G.)

**Keywords:** semiflexible polymer, active Brownian particle, active polymer, polymer conformations, polymer dynamics

## Abstract

The conformational properties of flexible and semiflexible polymers exposed to active noise are studied theoretically. The noise may originate from the interaction of the polymer with surrounding active (Brownian) particles or from the inherent motion of the polymer itself, which may be composed of active Brownian particles. In the latter case, the respective monomers are independently propelled in directions changing diffusively. For the description of the polymer, we adopt the continuous Gaussian semiflexible polymer model. Specifically, the finite polymer extensibility is taken into account, which turns out to be essential for the polymer conformations. Our analytical calculations predict a strong dependence of the relaxation times on the activity. In particular, semiflexible polymers exhibit a crossover from a bending elasticity-dominated dynamics to the flexible polymer dynamics with increasing activity. This leads to a significant activity-induced polymer shrinkage over a large range of self-propulsion velocities. For large activities, the polymers swell and their extension becomes comparable to the contour length. The scaling properties of the mean square end-to-end distance with respect to the polymer length and monomer activity are discussed.

## 1. Introduction

A distinctive characteristic of active matter is the conversion of internal chemical energy into, or utilization of energy from the environment for, directed motion [1,2,3,4,5,6,7,8,9]. The spectrum of biological active systems is wide and ranges from the macroscopic scale of flocks of birds and mammalian herds [3], the cytoskeleton in living cells [2,5,10,11,12,13,14,15,16,17], down to moving bacteria [2,6,18] on the micrometer scale. Thereby, nature employs various propulsion strategies. Bacteria are typically propelled by helical flagella [6,18,19,20,21]. The actin filaments of the cytoskeleton are driven forward by molecular motors [5,14,15,16,17,22]. Alike, microtubules in motility assays are propelled by surface-bound dyneins [23]. For synthetic active particles, chemical or physical propulsion mechanism are exploited [24,25,26,27].

Various features are common to all active systems [28], and the challenge of a theoretical description is to find a suitable approach capturing these characteristics. Generically, the activity-induced hydrodynamic flow field of a microswimmer is described by a force dipole [1,29,30]. Experiments, theoretical calculations and computer simulations, e.g., for *Escherichia coli* bacteria [30,31,32,33,34] and *Chlamydomonas reinhardtii* algae [31,32,35,36] confirm such a description for the far-field flow. However, the near-field flow can be distinctively different from the flow field of a force dipole [31,32,34,35,36].

Microswimmers are often described as active Brownian particles (ABPs) [4,24,28,37,38,39,40,41,42], neglecting hydrodynamics. This minimal stochastic model already yields interesting propulsion and excluded volume-induced emerging structures [4,38,39,40,41]. Moreover, ABPs are an extremely useful model to unravel the out-of-equilibrium statistical features of active systems [43,44,45,46,47,48,49,50,51].

The properties of connected active particles, such as linear chains [28,52,53,54,55,56,57,58,59,60,61,62,63,64,65,66] or other arrangements [67], are particular interesting systems, because of the coupling of their conformational properties and propulsion. Similar to external forces, the intrinsic activity leads to significant conformational changes, as shown in [28,57,68]. In this context, we also like to mention the conformational modulations of polymers embedded in a bath of active Brownian particles [69,70]. Activity also affects other polymer properties. An example is the linear viscoelastic response of an entangled, isotropic solution of semiflexible polymers as a model systems for myosin-driven actin filaments [52]. Here, activity leads to novel time-dependent regimes of the shear modulus. Other aspects are emerging beat patterns [54], activity-induced ring closure [53,71], aggregation of individual polymers in two dimensions [57] and collective phenomena [55]. Moreover, the internal dynamics of active dumbbells [28] and polymers [56,71] has been addressed. The influence of hydrodynamic interactions on the dynamical properties of active polymer properties have been analyzed in [59,60,62,72].

The (theoretical) analysis of the nonequilibrium behavior of flexible and semiflexible polymers, e.g., under shear flow [73,74,75,76,77,78] or during stretching [79,80,81,82,83,84,85,86,87,88,89,90,91,92,93], reveals the paramount importance of the finite polymer extensibility. We expect this intrinsic polymer property to be essential also for polymers comprising active monomers. Most theoretical studies have neglected finite polymer extensibility [56,68,71]. Only in the analytical treatment of the dynamics of an active dumbbell in [28] has the finite extensibility been taken into account and its fundamental importance for the dumbbell dynamics been demonstrated.

In this article, the conformational properties of flexible and semiflexible active Brownian polymers (ABPO) are studied analytically. Thereby, we consider a polymer composed of active Brownian particles, which are assembled in a linear chain. The diffusive motion of the propulsion velocity of the monomers is described by a Gaussian, but non-Markovian process. The emphasize is on the conformational properties due to the intimate coupling of the entropic polymer degrees of freedom and the activity of the monomers. We adopt the Gaussian semiflexible polymer model [82,94], which allows us to treat the problem analytically. As an important extension to previous studies, we account for the finite polymer extensibility and demonstrate that it strongly affects the out-of-equilibrium properties of an active polymer. Evaluation of the polymer relaxation times shows a drastic influence of that constraint on the polymer dynamics. In general, the relaxation times decrease with increasing activity, whereby the decline is more pronounced for stiffer polymers. Here, activity induces a transition from semiflexible-polymer behavior, determined by bending elasticity, to the entropy-dominated behavior of flexible polymers with increasing activity. Correspondingly, the conformational properties depend on activity. In the simpler case of flexible polymers, activity leads to their swelling over a wide range of activities. Thereby, the dependence on activity is very different from the theoretical prediction of a Rouse model [68]. Interestingly, semiflexible polymers exhibit an activity-induced shrinkage. However, for large activities the polymer conformations are ultimately comparable with those of flexible polymers. The shrinkage of active polymers in two dimensions has been observed by simulations in [68]. However, that shrinkage is due to excluded-volume effects and is unrelated to our observations for semiflexible polymers, where excluded-volume interactions are negligible.

Our theoretical considerations shed light on the nonequilibrium properties of semiflexible polymers and underline the importance of an adequate description already for moderate activities. Models without the constraint of a finite contour length, e.g., the standard Rouse model [95], would by no means be able to reproduce and capture the correct structural and dynamical aspects.

## 2. Model of Active Polymer

**Figure 1:**
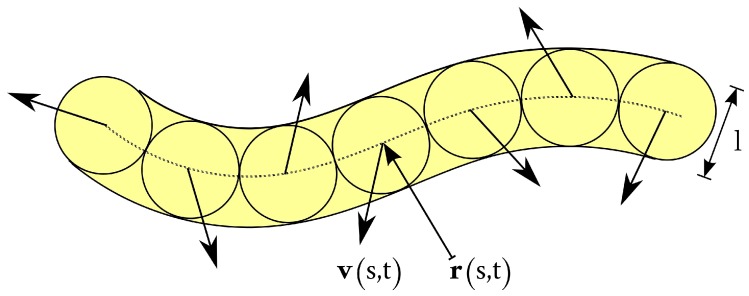
Model of the continuous semiflexible active polymer.

We adopt a mean-field model for a semiflexible polymer [82,94,96,97,98,99], which is denoted as Gaussian semiflexible polymer (GSFP), complemented by the activity of the monomers (GSFAP). We describe the GSFP as a continuous, differentiable space curve r(s,t), where *s* (−L/2≤s≤L/2) is the contour coordinate along the chain of length *L* and *t* is the time. Activity is added by assigning the self-propulsion velocity v(s,t) to every point r(s,t), as typical for active Brownian particles (cf. Figure 1) [6,7,8,38,39,41]. The equation of motion of the GSFAP is then given by the Langevin equation [78,100,101,102,103]:

(1) $$\begin{array}{c} \frac{\partial }{\partial t}r\left(s,t\right)=v\left(s,t\right)+\frac{1}{\gamma }\left(2\lambda k_{B}T\frac{\partial^{2}}{\partial s^{2}}r\left(s,t\right)-\epsilon k_{B}T\frac{\partial^{4}}{\partial s^{4}}r\left(s,t\right)+\Gamma \left(s,t\right)\right)\; \end{array}$$

with the boundary conditions:

(2) $$\begin{array}{c} {\left[2\lambda \frac{\partial }{\partial s}r\left(s,t\right)-\epsilon \frac{\partial^{3}}{\partial s^{3}}r\left(s,t\right)\right]}_{s=\pm L/2}=0\;,\;{\left[2\lambda_{0}\frac{\partial }{\partial s}r\left(s,t\right)\pm \epsilon \frac{\partial^{2}}{\partial s^{2}}r\left(s,t\right)\right]}_{s=\pm L/2}=0\; \end{array}$$

The terms with the second and fourth derivative in Equation (1) account for the entropic degrees of freedom and bending restrictions, respectively. Formally, the entropic part looks like a stretching energy due to harmonic bonds along the polymer contour with $\lambda k_{B}T$ and $\lambda_{0}k_{B}T$ as the Hookean spring constants [79,104] of the continuous chain. In the following, we will denote *λ* and $\lambda_{0}$ as stretching and *ϵ* as the bending coefficient. Note that *λ* and $\lambda_{0}$ are in general different due to the broken symmetry at the chain ends. The stochastic force Γ(s,t) is assumed to be stationary, Markovian and Gaussian with zero mean and the second moments:

(3) $$\begin{array}{c} \langle \Gamma_{\alpha }\left(s,t\right)\Gamma_{\beta }\left(s^{\prime },t^{\prime }\right)\rangle =2\gamma k_{B}T\delta_{\alpha \beta }\delta \left(s-s^{\prime }\right)\delta \left(t-t^{\prime }\right)\; \end{array}$$

where *T* is the temperature, $k_{B}$ the Boltzmann constant, *γ* the translational friction coefficient per length and α,β∈{x,y,z}. The Lagrangian multipliers *λ*, $\lambda_{0}$ and *ϵ* are determined by constraints [80,82]. In general, we find ϵ=3/4p and $\lambda_{0}=3/4$ for a polymer in three dimensions, where *p* is related to the persistence length $l_{p}$ via $p=1/2l_{p}$ [80,82], i.e., the bending coefficient $\epsilon =3l_{p}/2$ is solely determined by the persistence length, as is well known [103,105,106]. In Equation (1), we apply a mean-field value for the Lagrangian multiplier *λ*. Strictly, we expect the Lagrangian multiplier to depend on the contour coordinate for the active system, because, as shown in [76,78,80,82,83], *λ* strongly depends on the presence of an external force, i.e., λ=λ(s), since it is determined by the local inextensibility condition $\langle {\left(\partial r/\partial s\right)}^{2}\rangle =1$. However, in Equation (1), we neglected this aspect and assume that *λ* is constant along the polymer contour. Hence, we imply the global constraint of a finite contour length:

(4) $$\begin{array}{c} \int_{-L/2}^{L/2}\langle {\left(\frac{\partial r(s,t)}{\partial s}\right)}^{2}\rangle ds=L \end{array}$$

corresponding to a mean-field approach. As a consequence, the polymer conformations may be inhomogeneous along its contour as, e.g., in the stretching of the GSFP [82]. However, the full solution of a discrete free-draining polymer model with individual Lagrangian multipliers for every bond and bond angle [80,82,94] yields expectation values for global quantities, such as viscosity, which deviate only very little from those determined with the constraint (4) in the limit of a nearly continuous polymer. Hence, the solution of the equations of motion with the constraint (4) suffices for many practical purposes.

We regard the self-propulsion velocity v(s,t) as a non-Markovian stochastic process in time with the correlation function:

(5) $$\begin{array}{c} \langle v\left(s,t\right)\cdot v\left(s^{\prime },t^{\prime }\right)\rangle =v_{0}^{2}le^{-\gamma_{R}\left|t-t^{\prime }\right|}\delta \left(s-s^{\prime }\right)\; \end{array}$$

Here, $v_{0}$ is the magnitude of the propulsion velocity and $\gamma_{R}$ the damping factor of the rotational motion. The velocity correlation function arises, on the one hand, from the independent stochastic process for the propulsion velocity:

(6) $$\begin{array}{c} \frac{\partial }{\partial t}v\left(s,t\right)=-\gamma_{R}v\left(s,t\right)+\eta \left(s,t\right) \end{array}$$

where η(s,t) is a Gaussian and Markovian stochastic forces with zero mean and the second moment:

(7) $$\begin{array}{c} \langle \eta \left(s,t\right)\cdot \eta \left(s^{\prime },t^{\prime }\right)\rangle =4D_{R}v_{0}^{2}l\delta \left(s-s^{\prime }\right)\delta \left(t-t^{\prime }\right) \end{array}$$

in three dimensions; $D_{R}=\gamma_{R}/2$ is the rotational diffusion coefficient. On the other hand, the correlation function (5) also follows for the active force $\gamma v_{0}e\left(s,t\right)$, with a constant self-propulsion velocity $v_{0}$ and the unit vector ***e*** of the propulsion direction, where ***e*** performs a random walk according to [6,8,28,51]:

(8) $$\begin{array}{c} \frac{\partial }{\partial t}e\left(s,t\right)=\hat{\eta }\left(s,t\right)\times e\left(s,t\right) \end{array}$$

Here, $\hat{\eta }\left(s,t\right)$ is a Gaussian and Markovian stochastic process with zero mean and the second moment:

(9) $$\begin{array}{c} \langle \hat{\eta }\left(s,t\right)\cdot \hat{\eta }\left(s,t\right)\rangle =4D_{R}l\delta \left(s-s^{\prime }\right)\delta \left(t-t^{\prime }\right) \end{array}$$

Since we will need and apply only the correlation function (5) in the following, the exact nature of the underlying process is irrelevant and our considerations apply for both type of processes.

Note that the continuum representation of the semiflexible polymer requires introducing a length scale *l* in Equations (5) and (7). With a touching-bead model in mind for a discrete polymer, this minimum length corresponds to the bead diameter and bond length of that model (cf. Figure 1). Strictly speaking, *l* is a free parameter in the continuum model. For a flexible polymer, we regard $l=2l_{p}=1/p$ as the Kuhn length [107,108].

In the above description, we consider the velocity ***v*** as an intrinsic property of the active polymer. However, we may also consider ***v*** as an external stochastic process with an exponential correlation (colored noise) [6,8,28,71]. Such a correlated noise may be exerted by active Brownian particles on an embedded polymer [63,69,70].

## 3. Solution of Equation of Motion

To solve the equation of motion (1), we apply an eigenfunction expansion in terms of the eigenfunctions of the eigenvalue equation [76,100]:

(10) $$\begin{array}{c} \epsilon k_{B}T\frac{d^{4}}{ds^{4}}\varphi_{n}\left(s\right)-2\lambda k_{B}T\frac{d^{2}}{ds^{2}}\varphi_{n}\left(s\right)=\xi_{n}\varphi_{n}\left(s\right)\;. \end{array}$$

The resulting eigenfunctions are given by [76,100]:

(11) $$\begin{array}{cc} \varphi_{0}= & \sqrt{\frac{1}{L}} \end{array}$$

(12) $$\begin{array}{cc} \varphi_{n}\left(s\right)= & \sqrt{\frac{c_{n}}{L}}\left(\zeta_{n}^{\prime }\frac{\sinh\zeta_{n}^{\prime }s}{\cosh\zeta_{n}^{\prime }L/2}+\zeta_{n}\frac{\sin\zeta_{n}s}{\cos\zeta_{n}L/2}\right),\;n\;\mathrm{odd} \end{array}$$

(13) $$\begin{array}{cc} \varphi_{n}\left(s\right)= & \sqrt{\frac{c_{n}}{L}}\left(\zeta_{n}^{\prime }\frac{\cosh\zeta_{n}^{\prime }s}{\sinh\zeta_{n}^{\prime }L/2}-\zeta_{n}\frac{\cos\zeta_{n}s}{\sin\zeta_{n}L/2}\right),\;n\;\mathrm{even} \end{array}$$

with:

(14) $$\begin{array}{c} \zeta_{n}^{\prime 2}-\zeta_{n}^{2}=\frac{2\lambda }{\epsilon }\;,\;\xi_{0}=0\;,\;\xi_{n}=k_{B}T\left(\epsilon \zeta_{n}^{4}+2\lambda \zeta_{n}^{2}\right)\; \end{array}$$

The $c_{n}$s follow from the normalization condition, and the wave numbers $\zeta_{n}$ and $\zeta_{n}^{\prime }$ are determined by the boundary conditions (2). $\varphi_{0}$ describes the translational motion of the whole molecule.

Inserting the eigenfunction expansions:

(15) $$\begin{array}{c} r\left(s,t\right)=\sum_{n\;=\;0}^{\infty }\chi_{n}\left(t\right)\varphi_{n}\left(s\right),\;\;\Gamma \left(s,t\right)=\sum_{n\;=\;0}^{\infty }\Gamma_{n}\left(t\right)\varphi_{n}\left(s\right),\;\;\eta \left(s,t\right)=\sum_{n\;=\;0}^{\infty }\eta_{n}\left(t\right)\varphi_{n}\left(s\right),\;\;v\left(s,t\right)=\sum_{n\;=\;0}^{\infty }v_{n}\left(t\right)\varphi_{n}\left(s\right) \end{array}$$

into Equation (1) yields the equation of motion for the mode amplitudes $\chi_{n}$:

(16) $$\frac{d}{dt}\chi_{n}\left(t\right)=-\frac{1}{\tau_{n}}\chi_{n}\left(t\right)+v_{n}\left(t\right)+\frac{1}{\gamma }\Gamma_{n}\left(t\right)$$

with the relaxation times:

(17) $$\begin{array}{c} \tau_{n}=\frac{\gamma }{\xi_{n}}=\frac{\gamma }{k_{B}T\left(\epsilon \zeta_{n}^{4}+2\lambda \zeta_{n}^{2}\right)}\; \end{array}$$

The stationary-state solution of Equation (16) is:

(18) $$\begin{array}{c} \chi_{n}\left(t\right)=e^{-t/\tau_{n}}\int_{-\infty }^{t}e^{t^{\prime }/\tau_{n}}\left(v_{n}\left(t^{\prime }\right)+\frac{1}{\gamma }\Gamma_{n}\left(t^{\prime }\right)\right)dt^{\prime }\; \end{array}$$

The time correlation functions of the mode amplitudes, which are useful in the further analysis, are obtained as $\langle \chi_{n}\left(t\right)\cdot \chi_{m}\left(t^{\prime }\right)\rangle =\delta_{nm}\langle \chi_{n}\left(t\right)\cdot \chi_{n}\left(t^{\prime }\right)\rangle$, with [28]:

(19) $$\begin{array}{c} \langle \chi_{n}\left(t\right)\cdot \chi_{n}\left(t^{\prime }\right)\rangle =\left(\frac{3k_{B}T\tau_{n}}{\gamma }e^{-|t-t^{\prime }|/\tau_{n}}+\frac{v_{0}^{2}l\tau_{n}^{2}}{1-{\left(\gamma_{R}\tau_{n}\right)}^{2}}\left[e^{-\gamma_{R}\left|t-t^{\prime }\right|}-\gamma_{R}\tau_{n}e^{-|t-t^{\prime }|/\tau_{n}}\right]\right)\; \end{array}$$

## 4. Results

### 4.1. Center-of-Mass Motion

The center-of-mass position is given by [100,102]:

(20) $$\begin{array}{c} r_{cm}\left(t\right)=\frac{1}{L}\int_{-L/2}^{L/2}r\left(s,t\right)\;ds=\chi_{0}\left(t\right)\varphi_{0}\left(t\right)\; \end{array}$$

With the solution of Equation (16) for the zeroth’s mode:

(21) $$\begin{array}{c} \chi_{0}\left(t\right)=\chi_{0}\left(0\right)+\int_{0}^{t}\left(v_{n}\left(t^{\prime }\right)+\frac{1}{\gamma }\Gamma_{n}\left(t^{\prime }\right)\right)dt^{\prime }\; \end{array}$$

we obtain the center-of-mass mean square displacement:

(22) $$\begin{array}{c} \langle {\left(r_{cm}\left(t\right)-r_{cm}\left(0\right)\right)}^{2}\rangle =\frac{6k_{B}T}{\gamma L}t+\frac{2v_{0}^{2}l}{\gamma_{R}^{2}L}\left(\gamma_{R}t-1+e^{-\gamma_{R}t}\right)\; \end{array}$$

As for an active Brownian particle, the term linear in time on the right-hand side accounts for the translational Brownian motion [6]. As a generalization, the total friction coefficient γL appears. The second term represents the contribution of activity. Again, it is similar to the term appearing for ABPs, aside from the ratio L/l. We can identify the latter as the number of frictional sites or monomers *N* of diameter *l*, i.e., N=L/l. Then, N=1 corresponds to an ABP with the friction coefficient γl and N=2 to a dumbbell [28,109].

The long-time diffusion coefficient follows as:

(23) $$\begin{array}{c} D=\frac{k_{B}T}{\gamma L}\left(1+\frac{3v_{0}^{2}l\gamma }{\gamma_{R}k_{B}T}\right)=D_{L}\left(1+\frac{3Pe^{2}}{2\Delta }\right)\; \end{array}$$

with the diffusion coefficient $D_{L}=k_{B}T/\gamma L$ of a passive polymer, the Péclet number Pe and the ratio Δ of the diffusion coefficients [6,28,110]:

(24) $$\begin{array}{c} Pe=\frac{v_{0}}{D_{R}l}\;,\;\;\;\Delta =\frac{D_{T}}{D_{R}l^{2}}\; \end{array}$$

Here, we introduce the diffusion coefficient $D_{T}=k_{B}T/\gamma l$ as the diffusion coefficient of a segment of length *l* (cf. description of the model on page 3). In the following, we use the thermal translational and rotational diffusion coefficients of spherical particles of diameter *l* in solution, which yields Δ=1/3.

### 4.2. Lagrangian Multiplier: Stretching Coefficient

Inextensibility is a fundamental property of a polymer and determines its conformational and dynamical characteristics. Hence, we have to calculate the Lagrangian multiplier *λ* first in order to relate other polymer aspects to the constraint Equation (4). Insertion of the eigenfunction expansion (15) for the position r(s,t) into Equation (4) yields:

(25) $$\begin{array}{c} \sum_{n\;=\;1}^{\infty }\left(\frac{3k_{B}T}{\gamma }\tau_{n}+\frac{v_{0}^{2}l}{1+\gamma_{R}\tau_{n}}\tau_{n}^{2}\right)\int_{-L/2}^{L/2}{\left(\frac{d\varphi_{n}\left(s\right)}{ds}\right)}^{2}ds=L \end{array}$$

which determines the Lagrangian multiplier *λ*. In terms of the Péclet number $Pe=v_{0}/D_{R}l$ and Δ of Equation (24), this equation can be expressed as:

(26) $$\begin{array}{c} \sum_{n\;=\;1}^{\infty }\left(\frac{1}{{\hat{\xi }}_{n}}+\frac{Pe^{2}N^{3}}{9\Delta^{2}\left({\hat{\xi }}_{n}^{2}+\frac{2N^{3}}{3\Delta }{\hat{\xi }}_{n}\right)}\right)\int_{-1/2}^{1/2}{\left(\frac{d\varphi_{n}\left(x\right)}{dx}\right)}^{2}dx=1\; \end{array}$$

with the abbreviation:

(27) $$\begin{array}{c} {\hat{\xi }}_{n}=pL\mu {\left(\zeta_{n}L\right)}^{2}+\frac{1}{4pL}{\left(\zeta_{n}L\right)}^{4}\; \end{array}$$

Here, we introduce the Lagrangian multiplier *μ* via the relation λ=3pμ/2, i.e., *μ* is the ratio between the stretching coefficients of the active and the passive polymer. In the integral, we substituted *s* by x=s/L.

**Figure 2:**
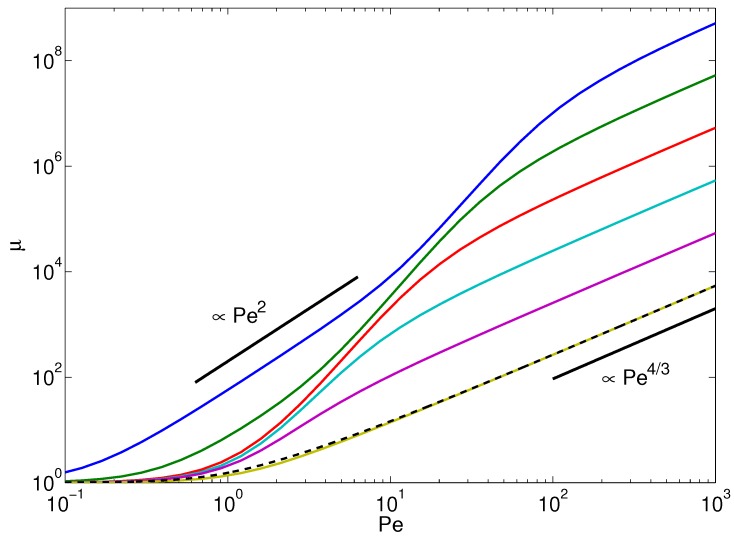
Normalized stretching coefficient (Lagrangian multiplier) μ=2λ/3p as a function of the Péclet number for the polymer bending stiffnesses $pL=10^{3}$, $10^{2}$, 10, 1, $10^{-1}$ and $10^{-2}$ (bottom to top). For the other parameters, we set $N=L/l=10^{3}$ and Δ=1/3. The dashed line for $pL=10^{3}$ represents the solution of the asymptotic Equation (31). The straight lines indicate the power-law dependencies $\mu ∼Pe^{2}$ for $pL<10^{-1}$ and Pe<1, and $\mu ∼Pe^{4/3}$ (cf. Equation (32)), respectively.

Figure 2 displays Lagrangian multipliers as a function of the Péclet number for various bending stiffnesses $pL=L/2l_{p}$ (at constant polymer length *L*, variation of pL corresponds to a variation of the polymer persistence length). Evidently, activity leads to an increase of the multiplier *μ* with increasing Pe. Thereby, semiflexible polymers with pL≲10 exhibit a pronounced dependence on Pe already for moderate Péclet numbers. In the limit Pe→0, the multiplier assumes the value of a passive polymer μ=1. Over the considered range of Péclet numbers, the curves exhibit the asymptotic dependence $\mu ∼Pe^{4/3}$ for large Pe, independent of the polymer stiffness. For polymers with pL≲10, an intermediate regime appears, where $\mu ∼Pe^{\kappa }$, with κ>3. Very stiff polymers ($pL<10^{-1}$) even exhibit another power-law regime for small Pe, where $\mu ∼Pe^{2}$. The various activity-induced features reflected in the Lagrangian multiplier imply pronounced effects on the conformations and internal dynamics of an active polymer.

*Flexible-polymer limit*: An analytical solution of Equation (25) can easily be obtained for a flexible polymer, where pL≫1. In this case, the wavenumbers are given by $\zeta_{n}=n\pi /L$, and the eigenfunctions reduce to trigonometric functions [100], such that:

(28) $$\begin{array}{c} \int_{-L/2}^{L/2}{\left(\frac{d\varphi_{n}\left(s\right)}{ds}\right)}^{2}ds\approx \zeta_{n}^{2}\; \end{array}$$

Hence, Equation (25) turns into:

(29) $$\begin{array}{c} \sum_{n\;=\;1}^{\infty }\left(\frac{3}{\epsilon \zeta_{n}^{2}+2\lambda }+\frac{v_{0}^{2}l\gamma^{2}}{k_{B}T\left(4\lambda^{2}k_{B}T+\epsilon \gamma \gamma_{R}\right)\zeta_{n}^{2}+2\lambda \gamma \gamma_{R}k_{B}T}\right)=L \end{array}$$

including modes up to order $n^{2}$. Evaluation of the sum yields:

(30) $$\frac{3L\sqrt{2\lambda }\coth\left(L\sqrt{2\lambda /\epsilon }\right)-3\sqrt{\epsilon }}{4\lambda \sqrt{\epsilon }}+\frac{\gamma lv_{0}^{2}L}{4\gamma_{R}k_{B}T\lambda }\left[\sqrt{\frac{2\gamma \gamma_{R}\lambda }{4k_{B}T\lambda^{2}+\epsilon \gamma \gamma_{R}}}\coth\left(L\sqrt{\frac{2\gamma \gamma_{R}\lambda }{4k_{B}T\lambda^{2}+\epsilon \gamma \gamma_{R}}}\right)-\frac{1}{L}\right]=L$$

or in terms of the Péclet number Pe and Δ (Equation (24)),

(31) $$\begin{array}{c} \frac{1}{\sqrt{\mu }}\coth\left(2pL\sqrt{\mu }\right)-\frac{1}{2pL\mu }+\frac{Pe^{2}}{6\mu \Delta }\left[\sqrt{\frac{\mu }{1+6\mu^{2}p^{3}l^{3}\Delta }}\coth\left(2pL\sqrt{\frac{\mu }{1+6\mu^{2}p^{3}l^{3}\Delta }}\right)-\frac{1}{2pL}\right]=1 \end{array}$$

The solution of this equation is compared to the exact solution of Equation (25) in Figure 2. Evidently, we find good agreement for pL≫1 and Pe≳10. Taking into account modes of order $n^{4}$ or even $n^{6}$ leads to a better agreement between the results of the two equations.

**Figure 3:**
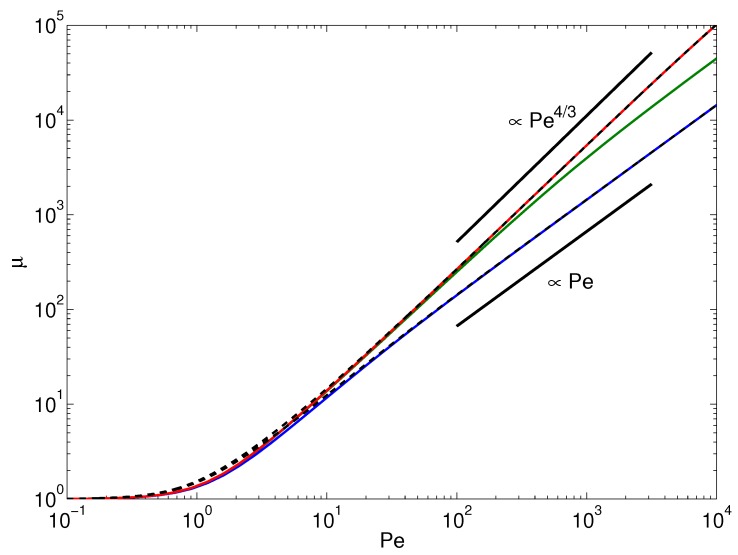
Normalized stretching coefficient μ=2λ/3p as function of the Péclet number for $pL=10^{1}$, $10^{2}$ and $10^{3}$ (bottom to top). In all cases, we set l=1/p, which corresponds to L/l=pL and Δ=1/3. The dashed lines represent the solution of the asymptotic Equation (31). The straight lines indicate the power-law dependencies $\mu ∼Pe^{4/3}$ for $N=10^{3}$ and μ∼Pe for N=10 (cf. Equations (32) and (33), respectively).

Equation (31) yields the following asymptotic dependencies:

- For a passive polymer, Pe=0 implies μ=1.
- In the limit pL→∞ and Pe<∞, i.e., 1≪μ<∞,
  (32) $$\begin{array}{c} \frac{1}{\sqrt{\mu }}+\frac{Pe^{2}}{\mu^{3/2}{\left(6pl\Delta \right)}^{3/2}}=1 \end{array}$$
  Hence, in the asymptotic limit pL→∞, $\mu ∼Pe^{4/3}/pl$ (cf. Figure 2 and Figure 3). Note that when we set l=1/p, i.e., identify *l* with the Kuhn length, *μ* is independent of the polymer length in the considered scaling regime. This is illustrated in Figure 3.
- For pL<∞ and Pe→∞, i.e., μ≫1,
  (33) $$\begin{array}{c} \frac{1}{\sqrt{\mu }}+\frac{Pe^{2}}{\mu^{2}}\frac{L}{54p^{2}l^{3}\Delta^{2}}=1 \end{array}$$
  which yields $\mu ∼Pe{\left(L/l\right)}^{3/2}/pL$ (cf. Figure 3). Here, there remains a polymer-length dependence for $l=l_{p}$, namely $\mu ∼Pe\sqrt{pL}$.

In the asymptotic limit Pe→∞, we find a crossover of the Lagrangian multiplier from the power-law dependence $\mu ∼Pe^{4/3}$ to μ∼Pe. In the latter regime, the Lagrangian multiplier depends on polymer length. The crossover behavior is illustrated in Figure 3. The figure presents results for flexible polymers of various lengths, where the Kuhn segment length is identified with *l*, i.e., pL=L/l. The power-law dependence $\mu ∼Pe^{4/3}$ is specific to the large number of internal degrees of freedom of a polymer. This applies to flexible, as well as semiflexible polymers. As is discussed in the next section, activity changes the properties of semiflexible polymers, and they exhibit flexible polymer behavior at large Péclet numbers. However, in the asymptotic limit Pe→∞, activity causes a stretching of the polymer and a crossover to the dependence μ∼Pe appears. The same relation is obtained for a finite-extensible active dumbbell, which lacks internal degrees of freedom [28]. Hence, the dynamical properties of active polymers are not only determined by the longest relaxation time, as is often the case for passive polymers, but the internal degrees of freedom play a much more significant role than for passive polymers.

### 4.3. Relaxation Times

The relaxation times (Equation (17)):

(34) $$\begin{array}{c} \tau_{n}=\frac{\gamma }{3k_{B}Tp}{\left(\mu \zeta_{n}^{2}+\frac{1}{4p^{2}}\zeta_{n}^{4}\right)}^{-1} \end{array}$$

depend via *μ* on the activity $v_{0}$ (or Pe). We like to emphasize once more that this is a consequence of the finite extensibility of a polymer [28]. Neglecting this intrinsic property implies μ=1, and the relaxation times are independent of the activity [68,71]. The presence of the factor *μ* gives rise to a particular dynamical behavior, specifically for semiflexible polymers.

In the limit of a flexible polymer, the relaxation times become:

(35) $$\begin{array}{c} \tau_{n}=\frac{\gamma L^{2}}{3\pi k_{B}Tp}\frac{1}{\mu n^{2}}=\frac{\tau_{R}}{\mu n^{2}}\; \end{array}$$

with the Rouse relaxation time $\tau_{R}=\gamma L^{2}/3\pi k_{B}Tp$ [95,100]. Since, μ⩾1 is a monotonically increasing function of Pe, activity accelerates the relaxation process and the relaxation times become shorter. However, the mode-number dependence is not affected.

**Figure 4:**
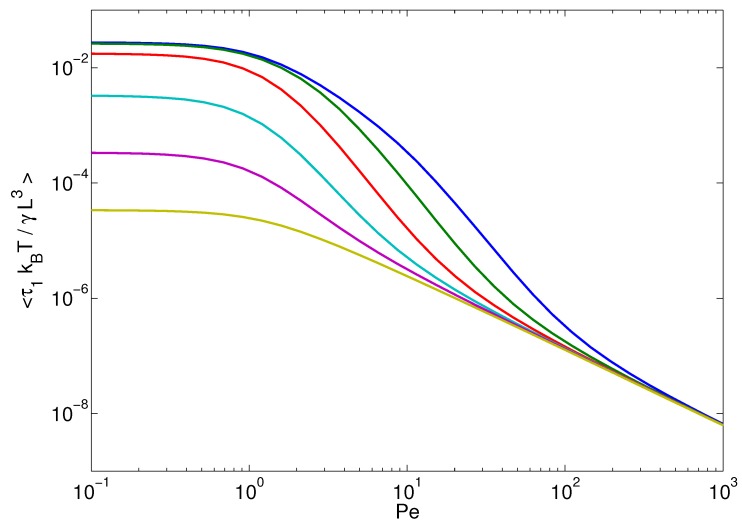
Longest polymer relaxation times as a function of the Péclet number for the bending stiffnesses (*L* is fixed) $pL=L/2l_{p}=10^{3}$, $10^{2}$, 10, 1, $10^{-1}$ and $10^{-2}$ (bottom to top). The other parameters are the same as in Figure 2.

The influence of activity on semiflexible polymers is much more substantial. For such polymers, pL<1, and the $\zeta^{4}$-dependence (bending modes) typically dominates the relaxation behavior. However, with increasing activity, and hence *μ*, the flexible modes ($\zeta_{n}^{2}$) in Equation (34) dominate over the bending modes. Thus, the contribution $\mu \zeta_{n}^{2}$ determines the relaxation behavior of the polymer for $n^{2}≲4{\left(pL\right)}^{2}\mu /\pi^{2}$. Only for larger modes, semiflexibility matters. As a consequence, starting from the large length-scale dynamics, activity induces a transition from semiflexible to flexible polymer behavior, which extends to smaller and smaller length scales with increasing Pe. This behavior is illustrated in Figure 4 for the longest polymer relaxation time $\tau_{1}$. For pL≫1, $\tau_{1}$ exhibits the predicted 1/μ behavior (cf. Equation (35)), with $\tau_{1}∼Pe^{-4/3}$ for large Pe. At Pe≲1, the relaxation times of the stiffer polymers are determined by the bending modes, and $\tau_{1}$ approaches the persistence-length and Pe independent value:

(36) $$\begin{array}{c} \tau_{1}=\frac{\gamma L^{3}}{36k_{B}T} \end{array}$$

with decreasing pL. The increase of *μ* with increasing Péclet number causes a decrease of the relaxation time $\tau_{1}$, and in the limit Pe≫1, the relaxation times assume the same asymptotic value of Equation (17) independent of the stiffness. Quantitatively, $\tau_{1}∼1/\mu$ as soon as $\mu \gg {\left(\pi /2pL\right)}^{-2}$. The latter is already satisfied for rather moderate Péclet numbers on the order of $Pe∼10^{1}-10^{2}$.

**Figure 5:**
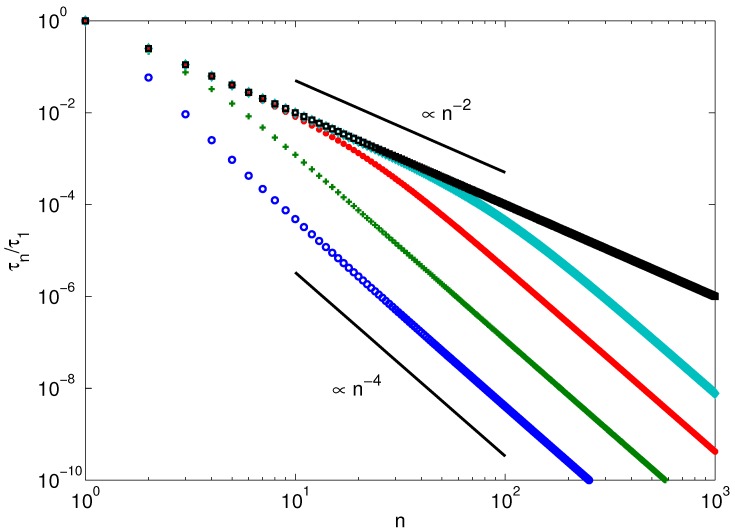
Mode-number dependence of the relaxation times of active polymers with $pL=10^{-2}$ for the Péclet numbers $Pe=10^{1}$, $3\times 10^{1}$, $10^{2}$ and $5\times 10^{2}$ (bottom to top). The black squares (top) show the mode-number dependence of a flexible polymer with $pL=10^{3}$. The other parameters are $N=10^{3}$ and Δ=1/3. The solid lines indicate the relations for flexible ($∼n^{-2}$) and semiflexible ($∼{\left(2n-1\right)}^{-4}$) polymers, respectively. $\tau_{1}$ is the longest relaxation time.

Figure 5 displays the dependence of the relaxation times $\tau_{n}$ of a stiff polymer on the mode number for various Péclet numbers. At low Pe, we find the well-known dependence $\tau_{n}/\tau_{1}∼{\left(2n-1\right)}^{-4}$ valid for semiflexible polymers [100,103,106]. With increasing Pe, the relaxation times increase, and for Pe≳50, the small-mode-number relaxation times exhibit the dependence $\tau_{n}/\tau_{1}∼n^{-2}$ of flexible polymers. At larger *n*, the relaxation times cross over to the semiflexible behavior again. However, the crossover point shifts to larger mode numbers with increasing activity. Taking the wavenumbers for flexible polymers, Equation (34) yields the condition $n>2pL\sqrt{\mu }/\pi$ for the dominance of bending modes. Hence, active polymers at large Péclet numbers appear flexible on large length and long time scales and only exhibit semiflexible behavior at small length scales.

### 4.4. Mean Square End-to-End Distance

To characterize the conformational properties of the polymers, we consider the mean square end-to-end distance $\langle r_{e}^{2}\rangle =\langle {\left(r\left(L/2\right)-r\left(-L/2\right)\right)}^{2}\rangle$, which is given by:

(37) $$\begin{array}{c} \langle r_{e}^{2}\rangle =4\sum_{n\;=\;1}^{\infty }\langle \chi_{2n-1}^{2}\rangle \varphi_{2n-1}^{2}\left(L/2\right) \end{array}$$

in terms of the eigenfunction expansion (15), where:

(38) $$\begin{array}{c} \langle \chi_{n}^{2}\rangle =\frac{3k_{B}T}{\gamma }\tau_{n}+\frac{v_{0}^{2}l}{1+\gamma_{R}\tau_{n}}\tau_{n}^{2}\;. \end{array}$$

**Figure 6:**
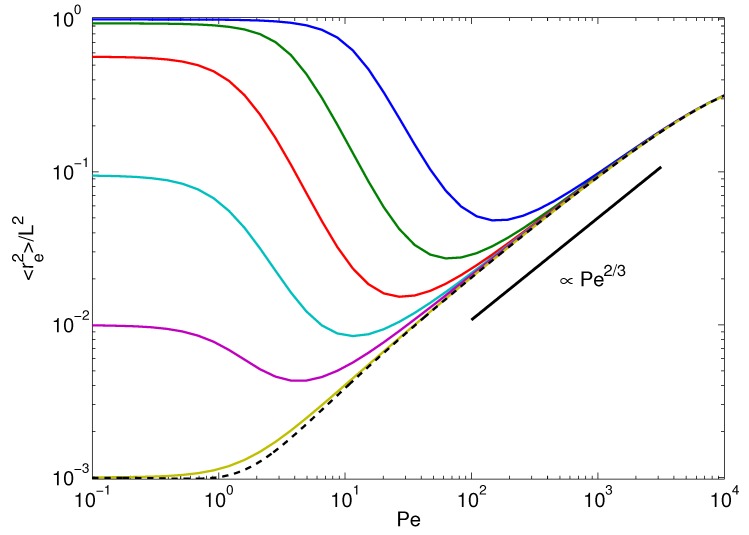
Mean square end-to-end distances as a function of the Péclet number for the polymer bending stiffnesses $pL=10^{3}$, $10^{2}$, 10, 1, $10^{-1}$ and $10^{-2}$ (bottom to top at $Pe=10^{-1}$). The other parameters are the same as in Figure 2. The dashed line represents the analytical solution of Equation (40) with the Lagrangian multiplier of Equation (31).

If the stretching coefficient *λ* and, hence, the relaxation times were independent of the activity, the average mean square mode amplitudes (38) would increase quadratically with the Péclet number for Pe→∞ (cf. the second term in the right-hand side of Equation (38)). Thus, the mean square end-to-end distance would increase quadratically with Pe [68]. As shown in Figure 6, the constraint of a constant contour length drastically changes the activity dependence of the polymer conformations. In the limit of a flexible polymer (bottom curve of Figure 6), $\langle r_{e}^{2}\rangle$ increases with increasing Péclet number as $Pe^{2/3}$ from the passive equilibrium value $\langle r_{e}^{2}\rangle =L/p$. The mean square end-to-end distances of passive polymers itself increases with increasing persistence length, until the limit $\langle r_{e}^{2}\rangle =L^{2}$ is reached for pL→0. For bending stiffnesses pL≲1 and Pe>1, activity causes a significant shrinkage of the polymer over a wide range of Péclet numbers. Above a certain Péclet number, the actual value depends on the stiffness, the polymer swells again, but now, similar to a flexible polymer, and the asymptotic value $\langle r_{e}^{2}\rangle =L^{2}/2$ is assumed for Pe→∞. This reflects the above-mentioned activity-induced transition from semiflexible to flexible-polymer behavior.

**Figure 7:**
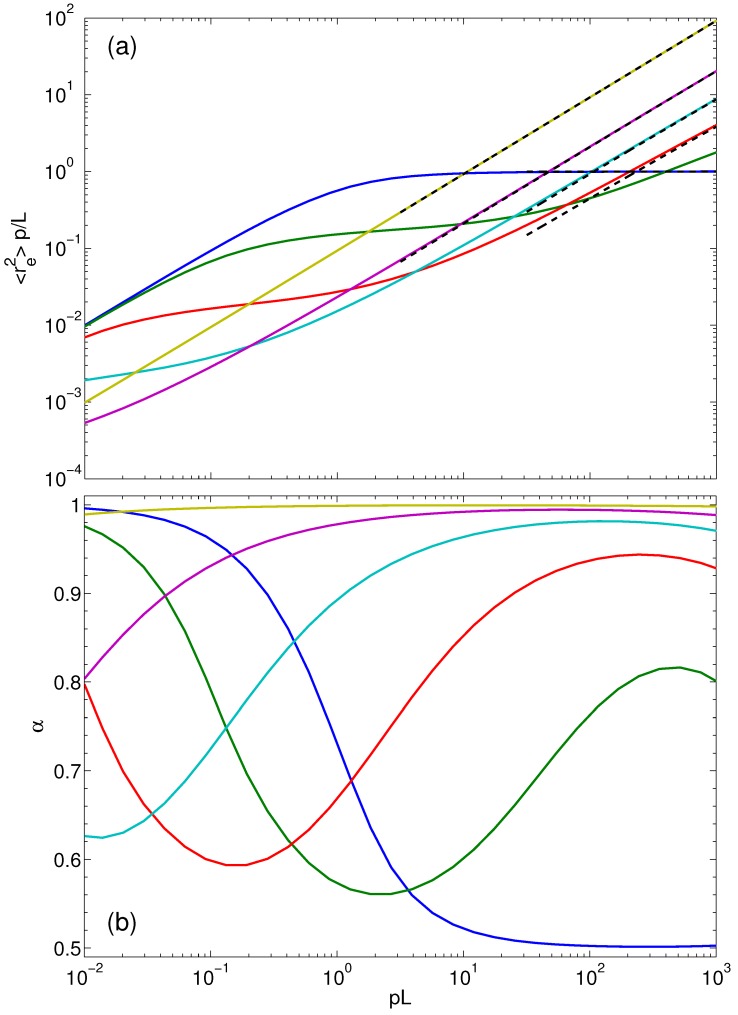
(**a**) Mean square end-to-end distances and (**b**) local slopes (Equation (39)) as function of the polymer length (pL) for the Péclet numbers Pe=0, 3, 10, 30, $10^{2}$ and $10^{3}$ (bottom to top at $pL=10^{3}$). The other parameters are the same as in Figure 2. The dashed lines in (**a**) represent the analytical solution of Equation (40) with the Lagrangian multiplier of Equation (31).

The scaling properties of $\langle r_{e}^{2}\rangle$ as a function of polymer length (pL) are illustrated in Figure 7a. In addition, Figure 7b shows the local slope:

(39) $$\begin{array}{c} \alpha =\frac{1}{2}\frac{d\log(\langle r_{e}^{2}\rangle )}{d\log(pL)} \end{array}$$

In the passive case Pe=0, $\langle r_{e}^{2}\rangle$ increases quadratically with increasing pL for pL<1 (α=1, rod-like scaling). In the limit pL≫1, the flexible Gaussian polymer scaling is obtained, where $\langle r_{e}^{2}\rangle =L/p$ (α=1/2), as is well know. In an active system, the local slope assumes the asymptotic value α=1 for pL→0, independent of the Péclet number Pe<∞. At a given Pe>0, the mean square end-to-end distance exhibits a monotonic progression with increasing pL, but the local slope is non-monotonic. Starting from the asymptotic value α=1, the local slope decreases first with increasing flexibility, i.e., pL, passes through a minimum, which depends on Pe, and increases again. This is illustrated in Figure 7b for Pe=3,10, and 30. The intermediate regime is rather broad, with local slopes almost as small as the value 1/2 for simple Gaussian polymers. In terms of scaling, we can identify a pL-regime for pL>1—the actual range depends on Pe—where *α* gradually increases with increasing Péclet number from the flexible polymer value α=1/2 to the rod limit α=1. In addition, (smaller) scaling regimes exist in the crossover region, which shift to smaller pL values with increasing Pe, with local slopes increasing from α=1/2 with increasing Péclet number. The slopes for Pe⩾3 decrease for large pL values. This is related to the selected density of active sites $N=10^{3}$ along the polymer. For $pL<10^{3}$, a polymer is stiff on the length scale p=1/l. In contrast, for $pL>10^{3}$, the polymer becomes flexible on lengths scales smaller than *l*, which gives rise to the decrease of the local slope.

*Flexible-polymer behavior*: Evaluation of Equation (37) in the limit of flexible polymers taking into account modes up to $n^{4}$, but neglecting all *ϵ* terms, yields:

(40) $$\begin{array}{c} \langle r_{e}^{2}\rangle =\frac{L}{p\mu }+\frac{Pe^{2}L}{6p\mu \Delta }\left[1-\frac{\sqrt{1+6p^{3}l^{3}\mu^{2}\Delta }}{pL\sqrt{\mu }}\tanh\left(\frac{pL\sqrt{\mu }}{\sqrt{1+6p^{3}l^{3}\mu^{2}\Delta }}\right)\right] \end{array}$$

This equation exhibits the asymptotic behaviors:

- For finite pL and Pe→∞, the argument of the hyperbolic tangent function becomes small, and Taylor expansion gives:
  (41) $$\begin{array}{c} \langle r_{e}^{2}\rangle \approx \frac{Pe^{2}L^{3}}{108p^{2}l^{3}\Delta^{2}\mu^{2}} \end{array}$$
  Insertion of the asymptotic behavior of Equation (33) for the Lagrangian multiplier yields $\langle r_{e}^{2}\rangle \overset{Pe\to \infty }{⟶}L^{2}/2$. Hence, the polymers assume nearly stretched conformations independent of the persistence length. This is visible in Figure 6.
- For Pe≫1, such that 1≪μ≪∞ and pL→∞, the argument of the hyperbolic tangent function becomes large. By setting the hyperbolic tangent to unity, we obtain:
  (42) $$\begin{array}{c} \langle r_{e}^{2}\rangle \approx \frac{L}{p\mu }\left(1+\frac{Pe^{2}}{6\Delta }\right) \end{array}$$
  Insertion of the asymptotics of Equation (32) for the stretching coefficient yields $\langle r_{e}^{2}\rangle \approx lLPe^{2/3}$. This dependence on the Péclet number is shown in Figure 6 for the polymer with $pL=10^{3}$.

## 5. Summary and Conclusions

We have presented an analytical approach to study the conformational and dynamical properties of active semiflexible polymers. We have adopted a continuum representation of a polymer with a certain number of active segments. Each of the segments is considered as an active Brownian particle whose orientation changes independently in a diffusive manner. Alternatively, the active random process can be considered as an additional external correlated (colored) noise acting on the polymer [6,8,28,71]. Active polymers have been considered before, both theoretically and by simulations [52,53,56,57,68,71]. As an important extension of the previous studies, we have taken into account the finite polymer extensibility due to its finite contour length. As has been shown, this constraint changes the dynamical behavior of active dumbbells drastically [28]. Taking into account the constraint by a Lagrangian multiplier leads to a linear equation, which is analytically tractable.

Evaluation of the polymer relaxation times shows a major influence of the finite contour length on the polymer dynamics. Models without such a constraint, e.g., the standard Rouse model [95], would not be able to reproduce and capture the correct dynamics, as reflected in the strong dependence of the stretching coefficient (Lagrangian multiplier) on the Péclet number already for moderate Pe values. In particular, the relaxation times decrease with increasing activity (Péclet number). Thereby, the influence of activity on stiff polymers is much more severe. Here, activity induces a transition from semiflexible-polymer behavior, characterized by bending modes, to flexible-polymer behavior, characterized by stretching modes, with increasing activity. Thereby, the affected length scale depends on the activity. For activities Pe≳20, the large length-scale and low-mode number properties are altered. With increasing Pe, an increasing number of modes and hence smaller length scales are affected. Due to the continuous nature of the considered polymer model, the (very) small-scale properties will always be dominated by bending modes.

The effect on the relaxation times translates to the conformational properties. In the simpler case of flexible polymers, activity leads to a monotonous swelling of the polymers over a wide range of Péclet numbers in a power-law manner, which is dictated by the constraint. Hence, our theoretical prediction is very different from the relation $\langle r_{e}^{2}\rangle ∼Pe^{2}$ of a Rouse model derived in [68] for any flexibility and Péclet number. For semiflexible polymers, with pL≲10, activity leads to shrinkage over a wide, stiffness-dependent range of Péclet numbers. At large Pe, the polymer conformations are comparable to those of flexible polymers. An activity-induced shrinkage of semiflexible passive polymers embedded in a fluid of ABPs has been observed in simulations of two-dimensional systems [69,70], in qualitative agreement with our theoretical predictions. This supports the equivalence between intramolecular activity and the impact of external colored noise on the properties of semiflexible polymers (cf. Section 2).

The simulation studies of [68] for two-dimensional ABPO predict an activity-induced shrinkage of self-avoiding polymers. These kinds of shrinkage may be particular for 2D ABPS in combination with self-avoidance. As stated in [68], the polymer shrinkage at moderate Péclet numbers can be attributed to activity-induced encaging by neighboring ABPs. The particular relevance of excluded-volume interactions in 2D systems is also reflected in other studies, e.g., in References [57,69,70]. The activity-induced shrinkage of our 3D semiflexible polymers is of different origin. Here, self-avoidance does not play any role. In general, self-avoidance is less important in 3D than in 2D systems. Nevertheless, we expect interesting collective dynamical effects in 3D systems based on our studies of suspensions of 3D ABPs [41]. Moreover, the 2D simulations of [68] suggest that the scaling relation of the mean square end-to-end distance with polymer length is unperturbed by the activity. However, this should only apply to (very) small Péclet numbers, as is evident from Figure 7, which suggest swelling of the polymer already for Pe≳1 and an activity-induced modified scaling behavior for large pL values. Note that the Péclet number of [68] is larger than ours due to the different definitions in terms of the translational and rotational diffusion coefficient, respectively. We definitely find for Pe>10 a wide crossover regime to the asymptotic scaling behavior of rod-like polymers, namely $\langle r_{e}^{2}\rangle ∼L^{2}$ (cf. Figure 7).

Our studies illustrated the usefulness of basic polymer models for the understanding of the complex interplay between polymer entropy, stiffness and activity. Extension of the current studies toward further dynamical properties and other propulsion preferences, e.g., along the tangent of the polymer contour, are under way.

Experimentally, chains of ABPs can be synthesized by linearly-connecting self-propelling Janus particles [7] by a flexible linker. A random distribution of linker sites on the colloid surface yields a random orientation of the propulsion directions of the individual “monomers”. The ensemble average over various realizations corresponds to our description.

## References

[1] Lauga E., Powers T.R. (2009). The hydrodynamics of swimming microorganisms. Rep. Prog. Phys..

[2] Ramaswamy S. (2010). The mechanics and statistics of active matter. Annu. Rev. Condens. Matter Phys..

[3] Vicsek T., Zafeiris A. (2012). Collective motion. Phys. Rep..

[4] Romanczuk P., Bär M., Ebeling W., Lindner B., Schimansky-Geier L. (2012). Active brownian particles. Eur. Phys. J. Spec. Top..

[5] Marchetti M.C., Joanny J.F., Ramaswamy S., Liverpool T.B., Prost J., Rao M., Simha R.A. (2013). Hydrodynamics of soft active matter. Rev. Mod. Phys..

[6] Elgeti J., Winkler R.G., Gompper G. (2015). Physics of microswimmers—single particle motion and collective behavior: A review. Rep. Prog. Phys..

[7] Bechinger C., Di Leonardo R., Löwen H., Reichhardt C., Volpe G., Volpe G. (2016). Active Brownian Particles in Complex and Crowded Environments. https://arxiv.org/abs/1602.00081.

[8] Marchetti M.C., Fily Y., Henkes S., Patch A., Yllanes D. (2016). Minimal model of active colloids highlights the role of mechanical interactions in controlling the emergent behavior of active matter. Curr. Opin. Colloid Interface Sci..

[9] Zöttl A., Stark H. (2016). Emergent behavior in active colloids. J. Phys. Condens. Matter.

[10] Nédélec F.J., Surrey T., Maggs A.C., Leibler S. (1997). Self-organization of microtubules and motors. Nature.

[11] Howard J. (2001). Mechanics of Motor Proteins and the Cytoskeleton.

[12] Kruse K., Joanny J.F., Jülicher F., Prost J., Sekimoto K. (2004). Asters, vortices, and rotating spirals in active gels of polar filaments. Phys. Rev. Lett..

[13] Bausch A.R., Kroy K. (2006). A bottom-up approach to cell mechanics. Nat. Phys..

[14] Jülicher F., Kruse K., Prost J., Joanny J.F. (2007). Active behavior of the cytoskeleton. Phys. Rep..

[15] Harada Y., Noguchi A., Kishino A., Yanagida T. (1987). Sliding movement of single actin filaments on one-headed myosin filaments. Nature.

[16] Schaller V., Weber C., Semmrich C., Frey E., Bausch A.R. (2010). Polar patterns of driven filaments. Nature.

[17] Prost J., Jülicher F., Joanny J.F. (2015). Active gel physics. Nat. Phys..

[18] Berg H.C. (2004). E. Coli in Motion.

[19] Scharf B. (2002). Real-time imaging of fluorescent flagellar filaments of rhizobium lupini H13-3: Flagellar rotation and ph-induced polymorphic transitions. J. Bacteriol..

[20] Copeland M.F., Weibel D.B. (2009). Bacterial swarming: A model system for studying dynamic self-assembly. Soft Matter.

[21] Kearns D.B. (2010). A field guide to bacterial swarming motility. Nat. Rev. Microbiol..

[22] Cordoba A., Schieber J.D., Indei T. (2014). A single-chain model for active gels I: active dumbbell model. RSC Adv..

[23] Sumino Y., Nagai K.H., Shitaka Y., Tanaka D., Yoshikawa K., Chate H., Oiwa K. (2012). Large-scale vortex lattice emerging from collectively moving microtubules. Nature.

[24] Howse J.R., Jones R.A.L., Ryan A.J., Gough T., Vafabakhsh R., Golestanian R. (2007). Self-motile colloidal particles: From directed propulsion to random walk. Phys. Rev. Lett..

[25] Volpe G., Buttinoni I., Vogt D., Kümmerer H.J., Bechinger C. (2011). Microswimmers in patterned environments. Soft Matter.

[26] Buttinoni I., Bialké J., Kümmel F., Löwen H., Bechinger C., Speck T. (2013). Dynamical clustering and phase separation in suspensions of self-propelled colloidal particles. Phys. Rev. Lett..

[27] Ten Hagen B., Kümmel F., Wittkowski R., Takagi D., Löwen H., Bechinger C. (2014). Gravitaxis of asymmetric self-propelled colloidal particles. Nat. Commun..

[28] Winkler R.G. (2016). Dynamics of flexible active Brownian dumbbells in the absence and the presence of shear flow. Soft Matter.

[29] Kim S., Karrila S.J. (1991). Microhydrodynamics: Principles and Selected Applications.

[30] Drescher K., Dunkel J., Cisneros L.H., Ganguly S., Goldstein R.E. (2011). Fluid dynamics and noise in bacterial cell-cell and cell-surface scattering. Proc. Natl. Acad. Sci. USA.

[31] Drescher K., Goldstein R.E., Michel N., Polin M., Tuval I. (2010). Direct measurement of the flow field around swimming microorganisms. Phys. Rev. Lett..

[32] Guasto J.S., Johnson K.A., Gollub J.P. (2010). Oscillatory Flows Induced by Microorganisms Swimming in Two Dimensions. Phys. Rev. Lett..

[33] Watari N., Larson R.G. (2010). The hydrodynamics of a run-and-tumble bacterium propelled by polymorphic helical flagella. Biophys. J..

[34] Hu J., Yang M., Gompper G., Winkler R.G. (2015). Modelling the mechanics and hydrodynamics of swimming *E. coli*. Soft Matter.

[35] Ghose S., Adhikari R. (2014). Irreducible representations of oscillatory and swirling flows in active soft matter. Phys. Rev. Lett..

[36] Klindt G.S., Friedrich B.M. (2015). Flagellar swimmers oscillate between pusher- and puller-type swimming. Phys. Rev. E.

[37] Peruani F., Schimansky-Geier L., Bär M. (2010). Cluster dynamics and cluster size distributions in systems of self-propelled particles. Eur. Phys. J. Spec. Top..

[38] Fily Y., Marchetti M.C. (2012). Athermal phase separation of self-propelled particles with no alignment. Phys. Rev. Lett..

[39] Bialké J., Speck T., Löwen H. (2012). Crystallization in a dense suspension of self-propelled particles. Phys. Rev. Lett..

[40] Redner G.S., Hagan M.F., Baskaran A. (2013). Structure and dynamics of a phase-separating active colloidal fluid. Phys. Rev. Lett..

[41] Wysocki A., Winkler R.G., Gompper G. (2014). Cooperative motion of active Brownian spheres in three-dimensional dense suspensions. EPL.

[42] Ten Hagen B., Wittkowski R., Takagi D., Kümmel F., Bechinger C., Löwen H. (2015). Can the self-propulsion of anisotropic microswimmers be described by using forces and torques?. J. Phys..

[43] Yang M., Ripoll M. (2014). A self-propelled thermophoretic microgear. Soft Matter.

[44] Solon A.P., Stenhammar J., Wittkowski R., Kardar M., Kafri Y., Cates M.E., Tailleur J. (2015). Pressure and phase equilibria in interacting active brownian spheres. Phys. Rev. Lett..

[45] Solon A.P., Fily Y., Baskaran A., Cates M.E., Kafri Y., Kardar M., Tailleur J. (2015). Pressure is not a state function for generic active fluids. Nat. Phys..

[46] Takatori S.C., Yan W., Brady J.F. (2014). Swim pressure: Stress generation in active matter. Phys. Rev. Lett..

[47] Maggi C., Marconi U.M.B., Gnan N., Di Leonardo R. (2015). Multidimensional stationary probability distribution for interacting active particles. Sci. Rep..

[48] Ginot F., Theurkauff I., Levis D., Ybert C., Bocquet L., Berthier L., Cottin-Bizonne C. (2015). Nonequilibrium equation of state in suspensions of active colloids. Phys. Rev. X.

[49] Bertin E. (2015). An equation of state for active matter. Physics.

[50] Speck T., Menzel A.M., Bialké J., Löwen H. (2015). Dynamical mean-field theory and weakly non-linear analysis for the phase separation of active Brownian particles. J. Chem. Phys..

[51] Winkler R.G., Wysocki A., Gompper G. (2015). Virial pressure in systems of spherical active Brownian particles. Soft Matter.

[52] Liverpool T.B., Maggs A.C., Ajdari A. (2001). Viscoelasticity of solutions of motile polymers. Phys. Rev. Lett..

[53] Sarkar D., Thakur S., Tao Y.G., Kapral R. (2014). Ring closure dynamics for a chemically active polymer. Soft Matter.

[54] Chelakkot R., Gopinath A., Mahadevan L., Hagan M.F. (2013). Flagellar dynamics of a connected chain of active, polar, Brownian particles. J. R. Soc. Interface.

[55] Loi D., Mossa S., Cugliandolo L.F. (2011). Non-conservative forces and effective temperatures in active polymers. Soft Matter.

[56] Ghosh A., Gov N.S. (2014). Dynamics of active semiflexible polymers. Biophys. J..

[57] Isele-Holder R.E., Elgeti J., Gompper G. (2015). Self-propelled worm-like filaments: Spontaneous spiral formation, structure, and dynamics. Soft Matter.

[58] Isele-Holder R.E., JÃ¤ger J., Saggiorato G., Elgeti J., Gompper G. (2016). Dynamics of self-propelled filaments pushing a load. Soft Matter.

[59] Laskar A., Singh R., Ghose S., Jayaraman G., Kumar P.B.S., Adhikari R. (2013). Hydrodynamic instabilities provide a generic route to spontaneous biomimetic oscillations in chemomechanically active filaments. Sci. Rep..

[60] Jayaraman G., Ramachandran S., Ghose S., Laskar A., Bhamla M.S., Kumar P.B.S., Adhikari R. (2012). Autonomous motility of active filaments due to spontaneous flow-symmetry breaking. Phys. Rev. Lett..

[61] Jiang H., Hou Z. (2014). Motion transition of active filaments: Rotation without hydrodynamic interactions. Soft Matter.

[62] Babel S., Löwen H., Menzel A.M. (2016). Dynamics of a linear magnetic “microswimmer molecule”. EPL.

[63] Kaiser A., Löwen H. (2014). Unusual swelling of a polymer in a bacterial bath. J. Chem. Phys..

[64] Valeriani C., Li M., Novosel J., Arlt J., Marenduzzo D. (2011). Colloids in a bacterial bath: Simulations and experiments. Soft Matter.

[65] Suma A., Gonnella G., Marenduzzo D., Orlandini E. (2014). Motility-induced phase separation in an active dumbbell fluid. EPL.

[66] Cugliandolo L.F., Gonnella G., Suma A. (2015). Rotational and translational diffusion in an interacting active dumbbell system. Phys. Rev. E.

[67] Küchler N., Löwen H., Menzel A.M. (2016). Getting drowned in a swirl: Deformable bead-spring model microswimmers in external flow fields. Phys. Rev. E.

[68] Kaiser A., Babel S., ten Hagen B., von Ferber C., Löwen H. (2015). How does a flexible chain of active particles swell?. J. Chem. Phys..

[69] Harder J., Valeriani C., Cacciuto A. (2014). Activity-induced collapse and reexpansion of rigid polymers. Phys. Rev. E.

[70] Shin J., Cherstvy A.G., Kim W.K., Metzler R. (2015). Facilitation of polymer looping and giant polymer diffusivity in crowded solutions of active particles. New J. Phys..

[71] Samanta N., Chakrabarti R. (2016). Chain reconfiguration in active noise. J. Phys. A Math. Theor..

[72] Laskar A., Adhikari R. (2015). Brownian microhydrodynamics of active filaments. Soft Matter.

[73] Dua A., Cherayil B.J. (2000). Chain dynamics in steady shear flow. J. Chem. Phys..

[74] Prabhakar R., Prakash J.R. (2006). Gaussian approximation for finitely extensible bead-spring chains with hydrodynamic interactions. J. Rheol..

[75] Dua A., Cherayil B.J. (2000). Effect of stiffness on the flow behavior of polymers. J. Chem. Phys..

[76] Winkler R.G., Keller S., Rädler J.O. (2006). Intramolecular dynamics of linear macromolecules by fluorescence correlation spectroscopy. Phys. Rev. E.

[77] Munk T., Hallatschek O., Wiggins C.H., Frey E. (2006). Dynamics of semiflexible polymers in a flow field. Phys. Rev. E.

[78] Winkler R.G. (2010). Conformational and rheological properties of semiflexible polymers in shear flow. J. Chem. Phys..

[79] Bird R.B., Curtiss C.F., Armstrong R.C., Hassager O. (1987). Dynamics of Polymer Liquids.

[80] Winkler R.G., Reineker P. (1992). Finite size distribution and partition functions of gaussian chains: Maximum entropy approach. Macromolecules.

[81] Marko J.F., Siggia E.D. (1995). Stretching DNA. Macromolecules.

[82] Winkler R.G. (2003). Deformation of semiflexible chains. J. Chem. Phys..

[83] Winkler R.G. (2010). Equivalence of statistical ensembles in stretching single flexible polymers. Soft Matter.

[84] Kierfeld J., Niamploy O., Sa-yakanit V., Lipowsky R. (2004). Stretching of semiflexible polymers with elastic bonds. Eur. Phys. J. E.

[85] Salomo M., Kegel K., Gutsche C., Struhalla M., Reinmuth J., Skokow W., Hahn U., Kremer F. (2006). The elastic properties of single double-stranded DNA chains of different lengths as measured with optical tweezers. Colloid Polym. Sci..

[86] Blundell J.R., Terentjev E.M. (2009). Stretching semiflexible filaments and their networks. Macromolecules.

[87] Lamura A., Winkler R.G. (2012). Semiflexible polymers under external fields confined to two dimensions. J. Chem. Phys..

[88] Hsu H.P., Binder K. (2012). Stretching semiflexible polymer chains: Evidence for the importance of excluded volume effects from Monte Carlo simulation. J. Chem. Phys..

[89] Radhakrishnan R., Underhill P.T. (2012). Models of flexible polymers in good solvents: Relaxation and coil–stretch transition. Soft Matter.

[90] Manca F., Giordano S., Palla P.L., Cleri F., Colombo L. (2012). Theory and Monte Carlo simulations for the stretching of flexible and semiflexible single polymer chains under external fields. J. Chem. Phys..

[91] Manca F., Giordano S., Palla P.L., Cleri F., Colombo L. (2013). Response to “Comment on ’Elasticity of flexible and semiflexible polymers with extensible bonds in the Gibbs and Helmholtz ensembles”’. J. Chem. Phys..

[92] Iliafar S., Vezenov D., Jagota A. (2014). In-plane force–extension response of a polymer confined to a surface. Eur. Polym. J..

[93] Alexeev A.V., Maltseva D.V., Ivanov V.A., Klushin L.I., Skvortsov A.M. (2015). Force-extension curves for broken-rod macromolecules: Dramatic effects of different probing methods for two and three rods. J. Chem. Phys..

[94] Winkler R.G., Reineker P., Harnau L. (1994). Models and equilibrium properties of stiff molecular chains. J. Chem. Phys..

[95] Doi M., Edwards S.F. (1986). The Theory of Polymer Dynamics.

[96] Bawendi M.G., Freed K.F. (1985). A Wiener integral model for stiff polymer chains. J. Chem. Phys..

[97] Battacharjee S.M., Muthukumar M. (1987). Statistical mechanics of solutions of semiflexible chains: A path integral formulation. J. Chem. Phys..

[98] Langowski J.B., Noolandi J., Nickel B. (1991). Stiff chain model—Functional integral approach. J. Chem. Phys..

[99] Ha B.Y., Thirumalai D. (1995). A mean-field model for semiflexible chains. J. Chem. Phys..

[100] Harnau L., Winkler R.G., Reineker P. (1995). Dynamic properties of molecular chains with variable stiffness. J. Chem. Phys..

[101] Winkler R.G., Harnau L., Reineker P. (1997). Distribution functions and dynamical properties of stiff macromolecules. Macromol. Theory Simul..

[102] Winkler R.G. (2006). Semiflexible polymers in shear flow. Phys. Rev. Lett..

[103] Winkler R.G. (2007). Diffusion and segmental dynamics of rod-like molecules by fluorescence correlation spectroscopy. J. Chem. Phys..

[104] Öttinger H.C. (1996). Stochastic Processes in Polymeric Fluids.

[105] Kratky O., Porod G. (1949). Röntgenuntersuchung gelöster Fadenmoleküle. Recl. Trav. Chim. PaysBas.

[106] Aragón S.R., Pecora R. (1985). Dynamics of wormlike chains. Macromolecules.

[107] Flory P.J. (1989). Statistical Mechanics of Polymer Chains.

[108] Rubinstein M., Colby R.C. (2003). Polymer Physics.

[B109-polymers-08-00304] 109.The Equation (10) of Reference 28 contains an error. The factor 2 in front of $v_{0}^{2}$ should be replaced by unity.

[110] Stenhammar J., Marenduzzo D., Allen R.J., Cates M.E. (2014). Phase behaviour of active Brownian particles: The role of dimensionality. Soft Matter.

